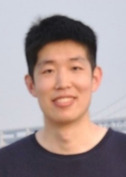# Light‐Driven Alcohol Splitting by Heterogeneous Photocatalysis: Recent Advances, Mechanism and Prospects

**DOI:** 10.1002/asia.202001312

**Published:** 2021-02-02

**Authors:** Zhigang Chai

**Affiliations:** ^1^ Department of Chemistry – Ångström Laboratory Uppsala University 75121 Uppsala Sweden

**Keywords:** Photocatalysis, LDAS, alcohol, hydrogen, visible light

## Abstract

Splitting of alcohols into hydrogen and corresponding carbonyl compounds, also called acceptorless alcohol dehydrogenation, is of great significance for both synthetic chemistry and hydrogen production. Light‐Driven Alcohol Splitting (LDAS) by heterogeneous photocatalysis is a promising route to achieve such transformations, and it possesses advantages including high selectivity of the carbonyl compounds, extremely mild reaction conditions (room temperature and irradiation of visible light) and easy separation of the photocatalysts from the reaction mixtures. Because a variety of alcohols can be derived from biomass, LDAS can also be regarded as one of the most sustainable approaches for hydrogen production. In this Review, recent advances in the LDAS catalyzed by the heterogeneous photocatalysts are summarized, focusing on the mechanistic insights for the LDAS and aspects that influence the performance of the photocatalysts from viewpoints of metallic co‐catalysts, semiconductors, and metal/semiconductor interfaces. In addition, challenges and prospects have been discussed in order to present a complete picture of this field.

## Graphical abstract

**Splitting alcohols into H_2_ and corresponding aldehydes or ketones with high selectivity under mild conditions** can be achieved by heterogeneous photocatalysis. Given that a variety of alcohols can be derived from biomass (e. g. lignocellulose), this approach is also regarded as one of the most sustainable routes for hydrogen production. In this review, recent advances in this field and mechanistic insights are discussed.

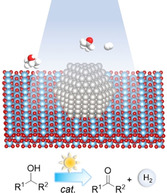

## 1 Introduction

**Scheme 1:**
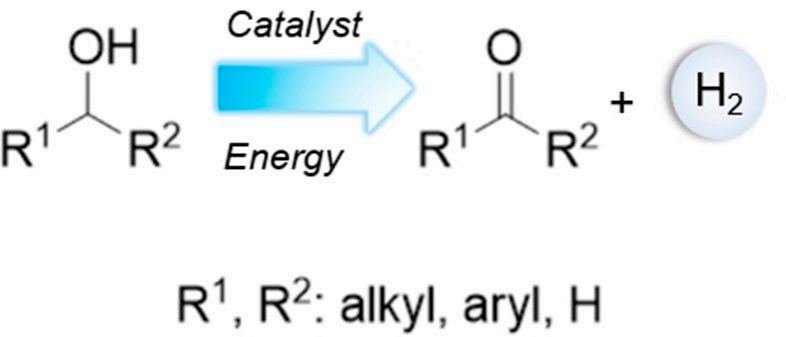
Alcohol splitting reaction.

Converting alcohols into carbonyl compounds (aldehydes and ketones) is one of the most important and fundamental reactions in synthetic chemistry. Despite the importance of this transformation, conventional alcohol oxidation methods suffer from several issues related to sustainability and selectivity. For example, alcohol oxidation is usually conducted utilizing the traditional metal‐based (Cr, Mn, Os) oxidants, hypervalent iodine reagents, and peroxide‐based oxidants; this process produces stoichiometric amount of waste and possesses low selectivity for some carbonyl compounds owing to over‐oxidation.[^1^, ^2^] To make the oxidation reactions cheaper and more environmentally friendly to the chemical industry, more benign and sustainable protocols have been developed utilizing air or molecular oxygen as the oxidant.[^3^, ^4^, ^5^, ^6^, ^7^] Although this reaction is an exothermic reaction, it is usually conducted at elevated temperature.[^3^, ^4^, ^5^, ^6^, ^7^] In some cases, the carbonyl compounds especially for aldehydes are prone to be further oxidized in the presence of O_2_.[^5^, ^7^] Another promising route which has received less attention is splitting of alcohols into hydrogen and corresponding carbonyl compounds (see Scheme 1), also called acceptorless alcohol dehydrogenation. The only by‐product, hydrogen, is a sustainable energy carrier due to its high energy capacity.[^8^, ^9^] It is worth noting that high selectivity of aldehydes or ketones can be achieved in this approach owing to the absence of oxidants.[^10^, ^11^, ^12^, ^13^, ^14^, ^15^, ^16^, ^17^, ^18^, ^19^, ^20^, ^21^, ^22^, ^23^, ^24^, ^25^, ^26^, ^27^, ^28^, ^29^, ^30^, ^31^, ^32^]

Alcohol splitting is an uphill reaction (endothermic), implying that energy is required to drive this reaction.[^10^, ^11^, ^12^, ^13^, ^14^, ^15^, ^16^, ^17^, ^18^, ^19^, ^20^, ^21^, ^22^, ^23^, ^24^, ^25^, ^26^, ^27^, ^28^, ^29^, ^30^, ^31^, ^32^] Conventional alcohol splitting reactions are usually conducted at equal or above 90 °C.[^10^, ^11^, ^12^, ^13^, ^14^, ^15^, ^16^, ^17^, ^18^, ^19^, ^20^, ^21^, ^22^, ^23^, ^24^] Alcohol splitting by electrolysis has also been reported, but an electrolyte and a complicated apparatus are needed.^[25]^ Therefore, developing an approach to perform alcohol splitting under mild conditions and in a readily available vessel is highly desirable.

Photocatalysis has been applied in a variety of chemical transformations with high selectivity under mild conditions.[^33^, ^34^, ^35^, ^36^, ^37^] Light‐driven alcohol splitting (LDAS) catalyzed by heterogeneous or homogeneous catalysts under mild conditions (irradiation of visible light, room temperature) and in a readily available vessel (e. g. flask) has been demonstrated.[^26^, ^27^, ^28^, ^29^, ^30^, ^31^, ^32^] In particular, LDAS catalyzed by heterogeneous photocatalysts can be conducted under neat condition; more importantly, the photocatalysts can be easily separated from the reaction mixtures and reused, greatly benefitting synthetic chemistry.[^29^, ^30^, ^31^, ^32^]

Notably, given that a variety of alcohols can be produced from transformation of biomass (e. g. lignocellulose),[^38^, ^39^, ^40^, ^41^, ^42^, ^43^, ^44^, ^45^] LDAS can also be regarded as a sustainable approach for hydrogen production.

## 2 Principles of Heterogeneous Photocatalysis

**Scheme 2:**
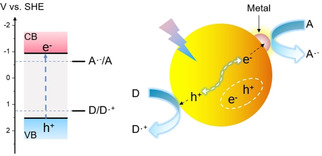
Processes involved in semiconductor photocatalysis (CB: conduction band, VB: valence band, A: electron acceptor, D: electron donor. Band‐edge positions of CdS are shown in the left panel).

Heterogeneous photocatalysis generally refers to semiconductor photocatalysis, principles of which has been reviewed.[^35^, ^46^, ^47^] Primary processes involved in the semiconductor photocatalysis are illustrated in Scheme 2. When a semiconductor is irradiated by a photon with energy equal or larger than the bandgap of the semiconductor, an electron is moved from the valence band (VB) to the conduction band (CB), simultaneously creating a positive hole in the VB. The photogenerated electron–hole pairs can recombine either directly or indirectly (e. g. via bulk or surface defects). In addition, the photogenerated electrons and holes can migrate to the surface of the semiconductor and react with electron acceptors and electron donors, respectively, resulting in photoredox reactions. From viewpoint of thermodynamics, the conduction band minimum (CBM) of the semiconductor must be more negative than the standard electrode potential of A ⋅ ^−^/A, while the valence band maximum (VBM) of the semiconductor must be more positive than the standard electrode potential of D/D ⋅ ^+^ as shown in Scheme 2.

Co‐catalysts, such as Pt, are often loaded on the semiconductor surface to suppress the recombination of photoexcited electron‐hole pairs via formation of the Schottky barrier at the metal/semiconductor interface.[^46^, ^48^] More importantly, co‐catalysts can significantly decrease the activation energies of the photoredox reactions and thus increase the reaction rates.

## 3 Light‐Driven Alcohol Splitting

### 3.1 Overview

To split an alcohol molecule, cleavages of O−H bond and α C−H bond are required. The corresponding carbonyl compounds of primary and secondary alcohol are aldehyde and ketone, respectively.

**Figure 1:**
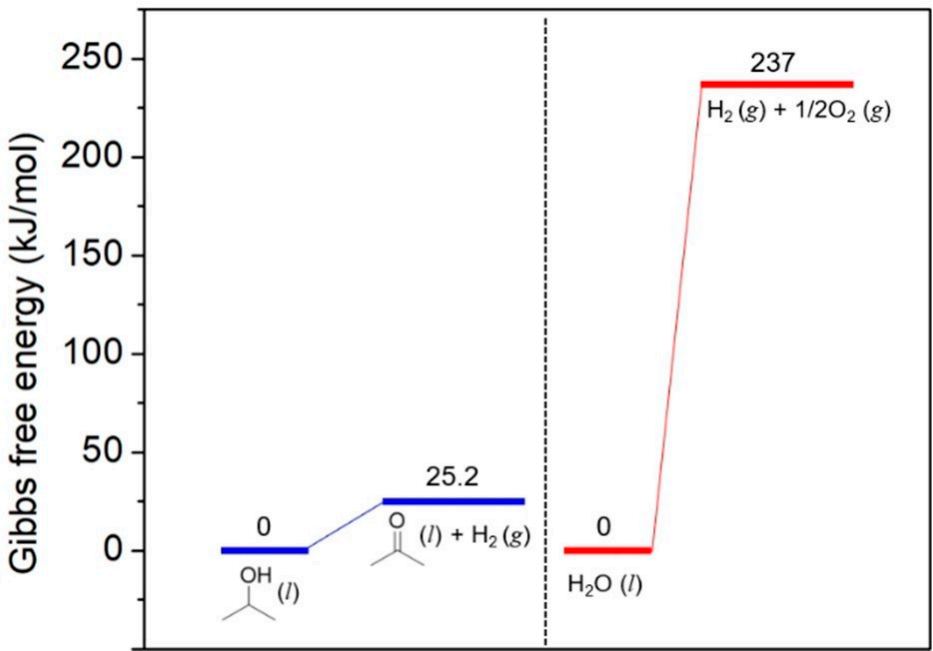
A comparison of alcohol splitting with overall water splitting.

The Gibbs free energy needed for driving alcohol splitting is usually in a range of 20–65 kJ mol^−1^ (298 K) depending on the alcohol and state of the substances. As shown in Figure 1, standard Gibbs free energy change for splitting of 2‐propanol is 25.2 kJ mol^−1^ which is much smaller than that required for overall water splitting (237 kJ mol^−1^).[^49^, ^50^] Accordingly, the photon energy required for alcohol splitting (several hundreds of meV) is much smaller than that required for overall water splitting (1.23 eV for one‐step photoexcitation),[^50^, ^51^, ^52^] implying full visible‐light regime and even part of near‐infrared light regime in the solar spectrum can be utilized for driving the LDAS reaction.

### 3.2 Evaluation of LDAS Reactions

Indicators which can evaluate the performance of photocatalysts in LDAS reactions are summarized here.

#### 3.2.1 Rate of H_2_ Production

As shown in Scheme 1, stoichiometric H_2_ is evolved along with formation of carbonyl compounds, therefore, rate of H_2_ production can be used to indicate the activity of the photocatalysts for the LDAS reaction. Note that different types of reactors and light sources can give different rates of H_2_ production even though employing the same photocatalyst; this makes it difficult to compare the results from different laboratories. Nonetheless, rate of H_2_ production normalized with respect to the weight of the photocatalyst is often reported. This normalized rate of H_2_ production ($r_{H_{2}}$ ) can be calculated as

(1) $$r_{H_{2}}=\frac{N\left(H_{2}\right)}{t\times m_{\mathrm{cat}.}}$$

where *N*(H_2_), *t*, and *m*_cat._ denote the number of evolved H_2_ molecules (in mol or mmol) in the alcohol splitting reaction, the irradiation time (in h), and the weight of the photocatalyst (in g), respectively; unit of $r_{H_{2}}$ is usually mmol h^−1^ g^−1^.

#### 3.2.2 Apparent Quantum Yield

For homogeneous photocatalysis, it is easy to calculate quantum yield owing to that the photons absorbed by the catalyst can be accurately measured. However, for heterogenous photocatalysis, light is not only absorbed but also scattered and reflected by the suspended heterogeneous photocatalysts;[^35^, ^53^] therefore, it is difficult to calculate quantum yield. In this case, apparent quantum yield (AQY), which is a lower limit of quantum yield, can be applied.^[35]^ AQY for a given photon energy is defined as:

(2) $$\mathrm{AQY}\;\left(\%\right)\;=\frac{n\times N_{p}}{N_{i}}\times 100\%$$

where *n* is the number of photons that is required to produce one molecule of product, *N*_p_ is the number of the products generated after reaction, and *N*_i_ is the number of incident photons.

For LDAS, two values of *n*, namely *n*=1 or *n*=2, have been assumed in practical calculations due to debates of the mechanism. Recently, Katsiev and co‐workers reported that the ratio between the evolved hydrogen and the absorbed photons is close to 1 in light‐driven ethanol splitting over Au clusters‐modified rutile TiO_2_(110) under ultra‐high vacuum;^[54]^ this result is also in line with the current‐doubling effect.[^55^, ^56^, ^57^, ^58^] Therefore, *n*=1, which means one photon absorbed by the photocatalyst generates one H_2_ molecule, is more likely on the basis of above results. Nonetheless, for purpose of comparison, the assumed value of *n* should be explicitly stated in the calculation of AQY.

#### 3.2.3 Solar Energy Conversion Efficiency

**Scheme 3:**
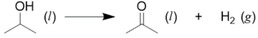
Splitting of 2‐propanol.

LDAS reaction can store energy (light energy) owing to the uphill nature of the reaction. In the case of splitting of 2‐propanol (Scheme 3), standard Gibbs free energy change for this reaction at 298 K is 25.2 kJ mol^−1^, which means splitting 1 mol of 2‐propanol results in storing 25.2 kJ of energy.

When a simulated solar spectrum is used as incident light, solar energy conversion efficiency (SECE) can be calculated:

(3) $$\mathrm{SECE}\;\left(\%\right)=\frac{\mathrm{Energy}\;\mathrm{stored}}{\mathrm{Energy}\;\mathrm{of}\;\mathrm{incident}\;\mathrm{solar}\;\mathrm{light}}\times 100\%=\frac{n_{H_{2}}\times \Delta G{}^{\circ }}{E_{\mathrm{in}}}\times 100\%$$

where $n_{H_{2}}$ , Δ*G*°, *E*_in_ denote amount of evolved H_2_, standard Gibbs free energy change of the reaction, and energy input of incident light, respectively.

#### 3.2.4 Turnover Number (TON) and Turnover Frequency (TOF)

TON and TOF can indicate the durability and activity of the photocatalyst, respectively. TON and TOF can be calculated as:

(4) $$\mathrm{TON}=\frac{N}{N_{\mathrm{sites}}}$$

(5) $$\mathrm{TOF}=\frac{N}{N_{\mathrm{sites}}\times t}$$

Where *N*, *N*_sites_, and *t* denote number of reactants that have been reacted, number of active sites, and reaction time, respectively. The unit of TOF is usually h^−1^ or s^−1^. A full description of TON and TOF in heterogenous catalysis can be found elsewhere.^[59]^

For LDAS, metallic co‐catalysts, such as Ni, Pt nanoparticles (NPs), are usually loaded on the semiconductors to achieve high efficiencies. In this case, the total number of surface sites of the metal NPs could be regarded as *N*_sites_, which can be determined by chemisorption of H_2_,[^60^, ^61^] or estimated according to the loading amount of the metal and averaged size of the metal NPs.[^3^, ^21^, ^31^]

#### 3.2.5 Conversion and Selectivity

Conversion indicates the fraction of reactants that has been converted after a certain time, it can be calculated as:

(6) $$\mathrm{Conversion}\;\left(\%\right)=\frac{N_{\mathrm{react}.}}{N_{\mathrm{total}}}\times 100\%$$

Where *N*_react._ represents the number of reactants that have been converted after a certain time, *N*_total_ is the total amount of the reactants before reaction.

Selectivity is important for LDAS because different products can be obtained owing to side reactions and/or existence of different kinds of OH groups in the alcohol molecule (e. g. glycerol molecule).

(7) $$\mathrm{Selectivity}\;\left(\%\right)=\frac{N_{\mathrm{target}}}{N_{\mathrm{react}.}}\times 100\%$$

Where *N*_target_ represents the number of reactants that is converted to the target product, *N*_react._ represents the total number of reactants that have been consumed.

## 4 A Brief Development History of LDAS

Report regarding light‐driven splitting of liquid alcohols can be tracked back to the pioneering work of Pichat and co‐workers in 1981.^[62]^ Since then, various semiconductor‐based photocatalysts have been developed, and most of them are metal‐modified titanium dioxide (TiO_2_) which are only active under irradiation of ultraviolet (UV) light.[^54^, ^62^, ^63^, ^64^, ^65^, ^66^, ^67^, ^68^, ^69^, ^70^, ^71^, ^72^, ^73^] One potential issue regarding using UV light is that undesirable by‐products might be obtained due to the high energy of the UV light. Furthermore, UV light only accounts for a very small fraction of the solar spectrum, about 4.6% (up to 400 nm, energy fraction). In contrast, visible light in the solar spectrum is far more abundant, about 47.8% (400–740 nm, energy fraction). Besides, most organic molecules absorb little or no visible light. Therefore, developing visible‐light active photocatalysts for LDAS is highly desired. Several visible‐light active semiconductors such as CdS,[^29^, ^30^, ^74^] CdS_x_Se_1‐x_,^[74]^ and graphitic carbon nitride[^31^, ^32^] have been modified with metal particles for catalyzing LDAS. In particular, it has been shown that Ni‐modified CdS (Ni/CdS) can efficiently split a variety of alcohols into H_2_ and corresponding carbonyl compounds in a stoichiometric manner under irradiation of visible light.^[29]^

## 5 Heterogeneous Photocatalysts for LDAS

**Figure 2:**
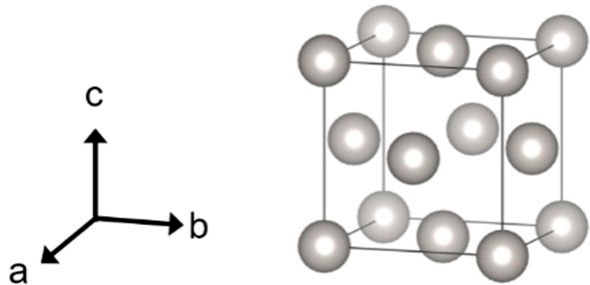
A unit cell of the face‐centered cubic (fcc) structure.

Loading metal particles on the surfaces of semiconductors can significantly enhance the activity of the pure semiconductors in LDAS, which could be attributed to the formation of the Schottky barrier[^46^, ^48^] and the catalytic properties of the metals. The metal particles, also called co‐catalysts, are transition metals such as Pt,[^32^, ^65^, ^66^, ^71^, ^74^, ^75^] Pd,[^32^, ^74^, ^75^] Ru,[^32^, ^75^] Au,[^67^, ^69^] Ni.[^29^, ^30^, ^63^, ^73^] Their crystal structures are usually face‐centered cubic (fcc) structures (Figure 2).

Rather than compiling all photocatalysts from the relevant literature, several representative semiconductor‐based photocatalysts for LDAS are summarized according to the type of the co‐catalysts.

### 5.1 Noble Metal as Co‐catalyst

**Figure 3:**
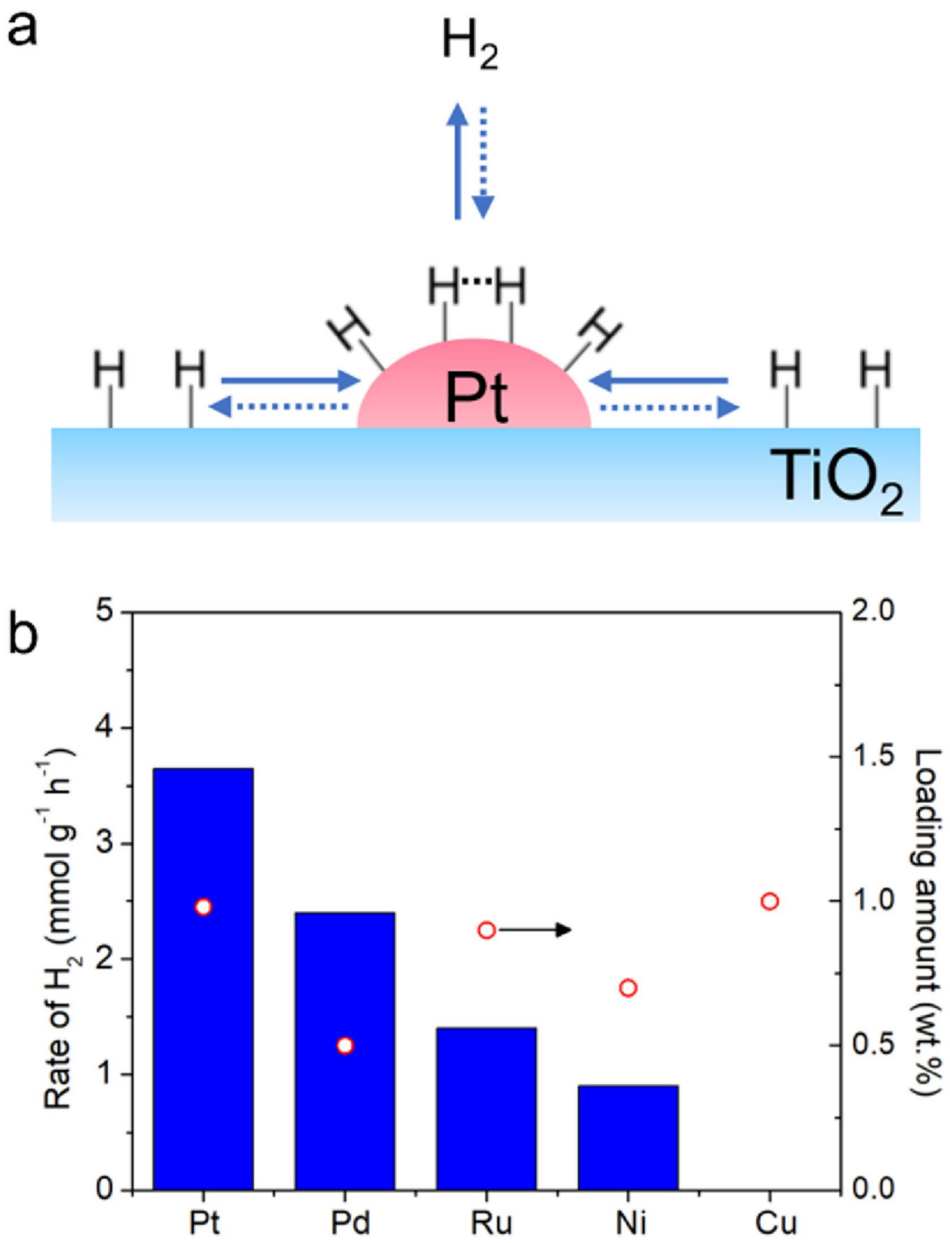
Schematic illustration of reverse and forward spillover in Pt/TiO_2_. The solid arrow and dashed arrow depict reverse spillover and forward spillover, respectively. (b) Rate of H_2_ from 2‐propanol over different metal‐loaded TiO_2_ (125 W high‐pressure Hg lamp), right y‐axis is loading amount of the metal. (b) was plotted using data from ref. [75].

Ichou and co‐workers studied the performance of Pt/TiO_2_ in 2‐propanol splitting in gas phase under UV irradiation. They prepared Pt/TiO_2_ by impregnation and subsequent reduction at 400 °C in H_2_. They found that migration of hydrogen towards Pt particle (reverse spillover, Figure 3a) was a rate‐limiting step when the loading amount of Pt was low. Forward spillover of hydrogen from Pt to TiO_2_ was not observed at room temperature because rate of the reaction was not decreased when partial pressure of H_2_ was up to 300 torrs. As shown in Figure 3b, they found that other metals modified TiO_2_ prepared by the same method also exhibited activity except Cu‐loaded TiO_2_.^[75]^

Murdoch and co‐workers investigated the effect of Au loading and particle size on light‐driven ethanol splitting over Au/TiO_2_. They found that Au particle size has no influence on the rate of H_2_ evolution over the 3–12 nm range. Interestingly, Au particles of similar size modified anatase nanoparticles exhibited a rate two orders of magnitude higher than that measured for Au modified rutile nanoparticles, they attributed this to the differences in the rates of electron–hole recombination between anatase and rutile.^[69]^

**Figure 4:**
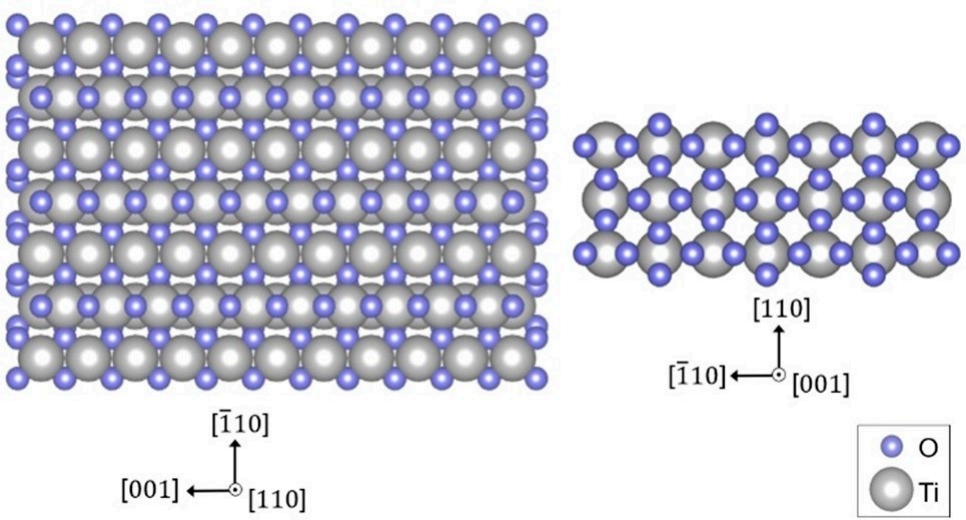
Atomic models of rutile TiO_2_ viewed along [110] and [001], respectively.

Rutile TiO_2_(110) is a readily available, one of the best understood crystalline materials (Figure 4);[^76^, ^77^, ^78^, ^79^] therefore, it has been widely used as the semiconductor to elucidate fundamental mechanisms in LDAS.[^54^, ^65^, ^71^, ^72^, ^73^, ^80^, ^81^, ^82^] Kollmannsberger and co‐workers investigated light‐driven splitting of methanol, ethanol, cyclohexanol, benzyl alcohol, and tert‐butanol on Pt cluster‐modified TiO_2_(110) single crystal under ultra‐high vacuum (UHV). They found that alcohols were converted to aldehydes or ketones along with the formation of stoichiometric H_2_ with the exception of tert‐butanol. A hole‐mediated α‐H abstraction of dissociatively adsorbed alcohol on the TiO_2_ surface and recombination of abstracted hydrogen at Pt clusters were proposed as the mechanism.^[65]^

Katsiev and co‐workers investigated the influence of Au cluster coverage on the light‐driven ethanol splitting by Au clusters‐modified rutile TiO_2_(110) under UHV. They observed a nonlinear increase of the H_2_ evolution rate with increasing gold coverage which is different from thermal catalytic reactions. They attributed this phenomenon to that increasing the cluster density within the Debye length will result in competitive trapping of the photogenerated electrons, which in turn decreases the reaction yield per cluster. Notably, they reported that the ratio between the evolved H_2_ and the number of absorbed photons under different photon flux is close to 1 and proposed that one absorbed photon can generate one H_2_ molecule in the current system. Quantum yield of the reaction under irradiation of UV light (310–400 nm) was reported to be about 2%.^[54]^

**Figure 5:**
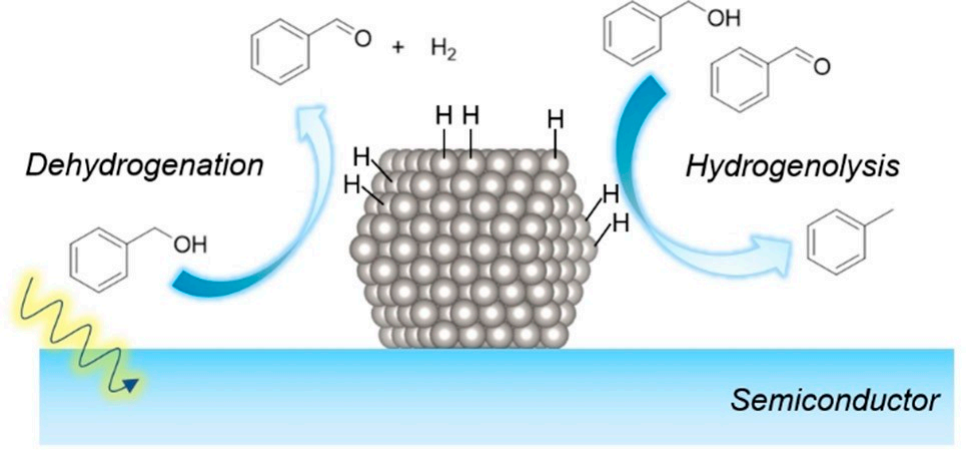
Dehydrogenation and hydrogenolysis of benzyl alcohol catalyzed by a metal‐modified semiconductor photocatalyst under irradiaton of visilbe light.

Ruberu and co‐workers studied metal‐modified semiconductor heterostructures for sunlight‐driven dehydrogenation and hydrogenolysis of benzyl alcohol (Figure 5).^[74]^ They found that Pt‐modified CdS favours dehydrogenation over hydrogenolysis (benzaldehyde as main product), whereas Pd‐modified CdS favours hydrogenolysis over dehydrogenation (toluene as main product).They also reported a transfer hydrogenation reaction in which glyceraldehyde is likely reduced by the photogenerated H_2_ or Pd−H hydride species on the surface of Pd‐modified semiconductor. They found that durability of the photocatalysts can be significantly enhanced after loading Pt or Pd NPs on the semiconductor surfaces.^[74]^ Notably, hydrogenolysis reactions have also been reported in conventional dehydrogenation of benzyl alcohol^[16]^ and 1‐phenylethanol^[48]^ over supported Pd catalysts.

Shibata and co‐workers reported that dehydrogenation reaction of primary aliphatic alcohols to aldehydes and H_2_ can be achieved under UV light irradiation over Au‐loaded TiO_2_ (Au/TiO_2_) which was prepared by an impregnation‐reduction method using TiO_2_ (P25) and sodium borohydride. AQY at 365 nm for dehydrogenation of 3‐phenylpropanol was determined to be 20% (*n*=2 in eqn. 2 was assumed). They investigated compatibility of functional groups for dehydrogenation of 3‐phenylpropanol and found the following results: 1) the efficiency of dehydrogenation of 3‐phenylpropanol remained unchanged in the presence of cyclohexanone or chlorobenzene; 2) the dehydrogenation reaction was slightly retarded in the presence of benzonitrile; 3) the dehydrogenation reaction was significantly inhibited by nitrobenzene and partial conversion of the nitrobenzene was observed.^[67]^

Sun and co‐workers reported that Pt nanoclusters‐modified graphitic carbon nitride (Pt/g‐C_3_N_4_) could split a variety of secondary alcohols into ketones with good to excellent yield under irradiation of simulated sunlight (300 W Xe lamp) using acetonitrile as solvent. They found that the Pt nanoclusters with a mean particle size of 2∼3 nm were loaded on g‐C_3_N_4_ after photodeposition. They investigated the mechanism of light‐driven splitting of diphenylmethanol via a series of control experiments and found that this reaction may proceed through a radical intermediate produced via a direct hole oxidation pathway.^[32]^

### 5.2 Non‐Noble Metal as Co‐catalyst

Nickel (Ni), which is earth‐abundant, has shown great potential in light‐driven alcohol splitting.[^29^, ^31^, ^63^] Owing to a negative value of the standard electrode potential of Ni^2+^/Ni (−0.26 V versus SHE), attention should be paid on preventing Ni from being oxidized by oxygen or water. Oxidation mechanism of Ni particles studied in an environmental transmission electron microscope has revealed that the surface of the metallic Ni particles can be oxidized at room temperature even at an O_2_ partial pressure of ∼10^−7^ mbar.^[83]^

Prahov and co‐workers studied hydrogen production from liquid methanol and 1‐propanol by Ni‐modified TiO_2_ under UV irradiation. They found that metallic Ni is the co‐catalyst that catalyzes the reaction.^[63]^

**Figure 6:**
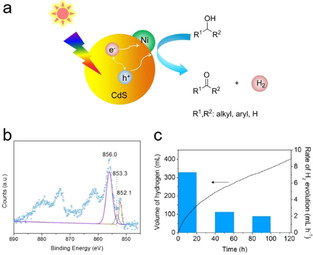
(a) A Schematic representation of visible light‐driven splitting alcohol over a Ni‐modified CdS (Ni/CdS) photocatalyst. (b) Ni 2p signal from XPS spectrum of Ni/CdS. (c) Long‐term light‐driven splitting of 2‐propanol. Reaction conditions: 2‐propanol (5 mL), Ni/CdS (6 mg), λ>420 nm (300 W Xe lamp), Ar, 20 °C. The bars denote the rate of hydrogen evolution averaged over 20 h of illumination. Reproduced with permission from ref. [29] (Copyright 2016, American Chemical Society).

Chai and co‐workers reported that Ni‐modified CdS (Ni/CdS) is capable of splitting a variety of alcohols into hydrogen and corresponding aldehydes or ketones in a stoichiometric manner under visible light irradiation (Figure 6a).^[29]^ They prepared the Ni/CdS by a photodeposition method in which methanol instead of water was used as solvent to prevent oxidation of Ni. Existence of metallic Ni was proved by transmission electron microscopy (TEM) and X‐ray photoelectron spectroscopy (XPS, Figure 6b). AQY values at 447 nm for splitting of methanol, ethanol, and 2‐propanol were found to be 38%, 46%, and 48%, respectively (*n*=2 in eqn. 2 was assumed). As shown in Figure 6c, the photocatalyst can be very stable during light‐driven splitting of 2‐propanol and a continuous H_2_ evolution was observed during 5 days (121 h). They reported a moderate conversion of 2‐propanol (25% after 121 h basing on the amount of the evolved H_2_), a high TON (44 465 after 121 h) and a high TOF (1222 h^−1^ for the first 6 h) for the visible light‐driven splitting of liquid 2‐propanol. In addition, a variety of alcohols including aromatic, cyclic and long‐chain aliphatic alcohols can be dehydrogenated to be corresponding carbonyl compounds with excellent selectivity by Ni/CdS under irradiation of visible light.^[29]^

Owing to the relatively negative value of the standard electrode potential of Ni^2+^/Ni (−0.26 V versus SHE) and fast recombination of the photogenerated electrons and holes of the semiconductor, the rate of photodeposition of Ni on the semiconductor surface can be very slow.^[31]^ To accelerate the photodeposition of Ni and simultaneously increase the dispersion of Ni, Chai and co‐workers reported a sequential photodeposition method to fabricate Ni−Ag nanostructure‐modified graphitic carbon nitride.^[31]^ They found that most of the Ni NPs were deposited on the surface of Ag owing to its ability of trapping the photoexcited electrons. They also reported that rate of solar‐driven splitting of ethanol over Ni−Ag nanostructure‐modified graphitic carbon nitride (Ni−Ag/CN_x_) was 4.3 times higher than that over Ni‐modified graphitic carbon nitride (Ni/CN_x_) owing to the smaller size of Ni particles and higher loading amount of Ni for Ni−Ag/CN_x_. For ethanol splitting catalyzed by Ni−Ag/CN_x_, AQY at 410 nm was calculated to be 0.15% (*n*=1 in eqn. 2 was assumed). They attributed the lower efficiency of Ni−Ag/CN_x_ compared with Ni/CdS for light‐driven ethanol splitting to the poor crystallinity of the employed CN_x_.^[31]^ In situ diffuse reflectance infrared Fourier transform spectroscopy (DRIFTS) was also performed, and intensity of a new vibration band attributed to acetate species over the Ni/CN_x_ was found to be much higher than that over Ni−Ag/CN_x_ upon illumination;^[31]^ they attributed this to the possible formation of Ag−Ni surface‐alloy which can suppress adsorption of acetate species.^[31]^ Notably, a poison effect of acetic acid was also observed in thermal catalytic dehydrogenation of cyclododecanol by Ni/θ‐Al_2_O_3_.^[14]^

## 6 Mechanistic Insights

Reaction mechanism of LDAS catalyzed by semiconductor‐based photocatalysts is considerably complicated because it involves heterogeneous catalysis as well as interfacial electron transfer reactions. Adsorption of alcohol molecules on the surfaces of semiconductors, cleavage of the α C−H bond (interaction between alcohol species and the photogenerated holes) and roles of metal co‐catalysts are three crucial steps for understanding the mechanism.

### 6.1 Adsorption of Alcohols

Ichou and co‐workers reported that the influence of alcohol pressure on the rate of splitting of 2‐propanol over Pt/TiO_2_ exhibited a kinetics which could be described by Langmuir adsorption isotherm.^[75]^ Shimizu and co‐workers observed heterolytic dissociation of (CH_3_)_2_CHOD on the surface of Al_2_O_3_ by in‐situ IR spectroscopy.^[14]^ In addition, adsorption of methanol on rutile TiO_2_(110) single crystal has been intensively studied.[^84^, ^85^, ^86^, ^87^, ^88^] Farfan‐Arribas and Madix found that methanol adsorbed dissociatively on the rutile TiO_2_(110) surfaces at room temperature, forming methoxide and hydroxide groups. On the stoichiometric surface, the methoxy species was bonded to Ti^4+^ cation, and most methoxide groups recombined at low temperature (320 K) to form methanol.^[87]^ Liu and co‐workers have conducted the sum frequency generation vibrational spectroscopy (SFG‐VS) measurements and density function theoretical (DFT) calculations to elucidate the methanol adsorption state on rutile TiO_2_(110). Both molecular and dissociative adsorption states of methanol were resolved in the SFG vibrational spectra. They also reported that the adsorption state of methanol considerably depends on the coverage: at no more than one layer, both methanol and methoxy species can coexist on five‐coordinated Ti cation (Ti_5c_) sites, which corresponds to a partially dissociated structure; the fraction of methoxy gradually decreases when the second layer appears and even disappears at multilayer coverage.^[88]^ Zhang and co‐workers performed DFT+U calculations to investigate the whole dissociation process of methanol into formaldehyde with and without photogenerated holes on rutile TiO_2_(110). In their report, the O−H dissociation of methanol is heterolytic and is likely to be thermally driven; the presence of a hole has no promotion on the barrier and enthalpy change. In contrast, the subsequent cleavage of C−H bond is homolytic and is possibly to be driven by photon; both kinetics and thermodynamics can be enhanced when holes are involved.^[85]^

Basing on the above results, one can conclude that alcohols can adsorb on the surfaces of semiconductors in both molecular and dissociative forms. Ratio of these two forms may depend on the coverage of the alcohols, the nature of the alcohols (e. g. acid dissociation constant) and properties of the surfaces of the semiconductors.

### 6.2 Cleavage of the α C−H bond

Regardless of the adsorption state of alcohols on the surfaces of semiconductors, cleavage of the α C−H bond driven by a photogenerated hole has been widely proposed.[^29^, ^32^, ^54^, ^65^] In this mechanism, alcohol species reacts with the photogenerated hole, resulting in homolytic scission of the α C−H bond. The alcohol species is converted into a carbonyl compound or a radical species depending on the its adsorption state (see Section 6.4). The cleavage of the α C−H bond also generates a hydrogen atom which either injects an electron to the semiconductor or directly binds to the surface of the metallic co‐catalysts (see Section 6.4).

Kinetic isotope effect (KIE) can provide information about the rate‐limiting step.[^14^, ^15^, ^29^] Chai and co‐workers reported a KIE (*k*_H_/*k*_D_) value of 5.1 (293 K) when LDAS was performed using a CH_3_OH/CD_3_OH (1 : 1 molar ratio) mixture over Ni/CdS.^[29]^ An estimated difference in the activation energies calculated by the Arrhenius equation was determined to be 4.0 kJ mol^−1^, which is close to the difference in the activation energies for C−H and C−D bond cleavages (ca. 5 kJ mol^−1^ at 300 K),^[89]^ indicating that the cleavage of the α C−H bond of methanol is the rate‐limiting step. In conventional dehydrogenation of alcohol by heterogeneous catalysts, cleavage of the α C−H bond of the alcohol was also found to be involved in the rate‐limiting step.[^14^, ^17^, ^90^] It has been found that different kinds of alcohols exhibits different activity in LDAS over the same photocatalyst;^[29]^ the measured activity of alcohols roughly follows an order: activated (aromatic) alcohols>aliphatic alcohols; secondary alcohols>primary alcohols.^[29]^ This can be attributed to the differences in the bonding energies of α C−H bonds.

### 6.3 Roles of Metallic co‐catalysts

Roles of the metal particles in LDAS include: 1) forming the Schottky barrier,[^46^, ^48^] which can trap the photogenerated electrons and thus suppress recombination of the photogenerated electrons and holes; 2) catalyzing hydrogen evolution reaction; the metal particles collect hydrogen species through either reducing a proton by the trapped photogenerated electron or abstracting a hydrogen atom from an intermediate of the alcohol molecule; the metal particles release two hydrogen atoms as one H_2_ molecule;[^29^, ^75^] 3) thermal catalytic hydrogenation and/or hydrogenolysis owing to existence of metal hydride (M−H) species (see Section 7.2).^[74]^

### 6.4 Two Possible Mechanisms

**Figure 7:**
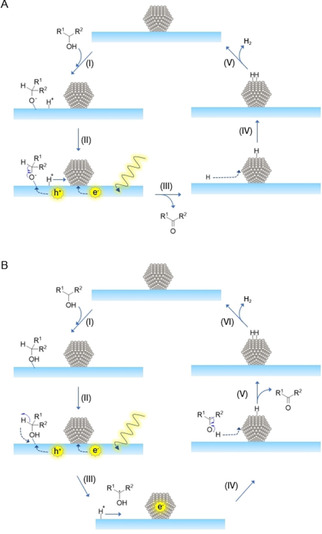
Two possible mechanisms for LDAS catalyzed by metal‐modified semiconductor photocatalysts (a cluster with fcc structure is used to represent the co‐catalyst).

On the basis of above‐mentioned results, it is proposed that alcohol molecules are adsorbed on the surface of the semiconductor in both molecular and dissociative states. Accordingly, two possible mechanisms which focus on interfacial electron transfer and neglect the specific surface structure of the semiconductor are proposed. For dissociative adsorption (mechanism A in Figure 7), in step (I), alcohol molecule adsorbs on the semiconductor surface through dissociating the OH bond (mainly heterolytic cleavage),^[85]^ forming adsorbed alkoxide anion and H^+^. For metal oxide‐based semiconductor, the alkoxide is usually coordinated with the metal cation and H^+^ is in the form of surface hydroxyl group.[^14^, ^86^, ^87^] In step (II), when the photocatalyst is irradiated by a photon with energy equal or bigger than bandgap of the semiconductor, photoexcited electron and hole are generated. The photoexcited electron is trapped in the metal particle and is supposed to reduce the proton that is migrated to the metal particle through reverse spillover,^[75]^ forming M−H hydride species;^[74]^ the photoexcited hole migrates to the surface of the semiconductor and reacts with the alkoxide anion, resulting in formation of a carbonyl compound and a hydrogen atom through a homolytic cleavage of the α C−H bond.[^29^, ^85^] In step (III), the carbonyl compound desorbs from the semiconductor surface. The hydrogen atom can undergo two reactions: 1) it can inject an electron to the CB of the semiconductor, forming a proton; the metallic co‐catalyst utilizes the electron to reduce a proton, producing a M−H species; 2) it can directly bind with the metallic co‐catalyst forming a M−H species. Both the two possible routes have the same result, that is, generating another M−H species (step (IV)). After releasing one H_2_ molecule via reverse dissociation of H_2_, the metal surface sites recover (step (V)).

For molecular adsorption (mechanism B in Figure 7), the alcohol adsorbs on the semiconductor surface via forming hydrogen bond and/or coordinating to the metal cation with an electron–pair of the oxygen atom in the alcohol (i. e. acting as a Lewis base). After the semiconductor absorb a photon with appropriate energy, the photoexcited electron is trapped in the metal particle, whereas the photoexcited hole migrates to the surface of the semiconductor and reacts with the alcohol molecule, resulting in formation of a hydrogen atom and a radical species via a homolytic cleavage of the α C−H bond. The hydrogen atom immediately reduces the photogenerated hole, producing a proton. The proton then migrates to the metal particle and is reduced by the photogenerated electron. The radical species can transform into the carbonyl compound, releasing a hydrogen atom which undergoes the two possible reactions as mentioned in the mechanism A.

Both the two possible mechanisms have a same net reaction, that is, one photon absorbed by the photocatalyst generates one H_2_ molecule and the corresponding carbonyl compound. This is in agreement with previously reported results[^54^, ^71^] and the current‐doubling effect.[^55^, ^56^, ^57^, ^58^]

**Scheme 4:**
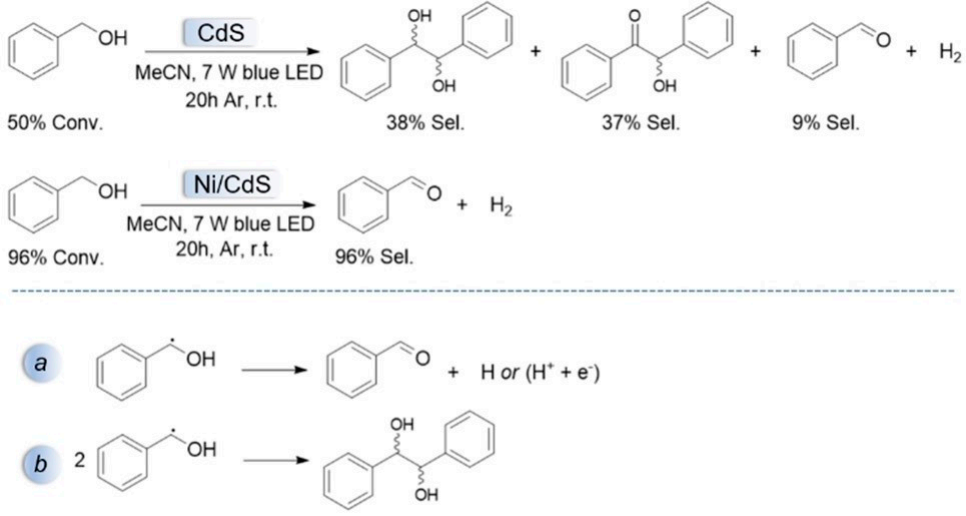
Top: Light‐driven splitting of benzyl alcohol over CdS and Ni/CdS, respectively. Conversion and selectivity values are from ref. [29]. Bottom: two possible reaction pathways for the benzyl radical species.

Notably, in mechanism B, the radical species may also undergo radical‐coupling reaction, forming a diol.[^29^, ^91^] This step is competitive with releasing hydrogen atom from the radical species.[^29^, ^91^] Chai and co‐workers reported that Ni loading on the surface of CdS can significantly increase the selectivity of the benzaldehyde in light‐driven splitting of benzyl alcohol.^[29]^ As shown in Scheme 4 (top panel), they found the flowing results: when pure CdS was employed, hydrobenzoin (38%) and benzoin (37%) as main products and a moderate conversion of benzyl alcohol (50%) were observed. In contrast, excellent selectivity of benzaldehyde (96%) and high conversion of benzyl alcohol (96%) were achieved when Ni/CdS was used.^[29]^ This could be attributed to that Ni NPs can significantly promote the hydrogen releasing step (pathway a, Scheme 4), thus inhibiting the radical‐coupling reaction (pathway b, Scheme 4).

Note that the alcohol and the carbonyl compound could also adsorb on the surface of metal particle. When M−H species exists, hydrogenation and/or hydrogenolysis can occur (see Section 7.2).^[74]^

### 6.5 Influence of Water

Previous studies have shown that water is not essential for the LDAS.[^29^, ^32^, ^65^, ^75^] In most cases, water is detrimental to the LDAS, for example, it can decrease the activity via competitive adsorption of water and alcohol;[^75^, ^92^, ^93^] it can deactivate Ni cocatalyst by oxidizing it.^[29]^ More importantly, if VBM of the semiconductor is positive enough, the valence holes can react with adsorbed water on the surface, forming ⋅ OH radicals.[^92^, ^94^] This is highly undesired for LDAS, because the ⋅ OH radicals possess very strong oxidation ability and can over‐oxidize the aldehydes to carboxylic acids[^95^, ^96^] and even degrade the carbonyl compounds,[^53^, ^96^] significantly decreasing the selectivity of the carbonyl compounds. Wang and co‐workers studied the competitive adsorption process between water and methanol on TiO_2_ through in situ sum frequency generation (SFG), a nonlinear spectroscopic technique. In their report, the methanol oxidation pathway, direct oxidation by photogenerated holes or indirect oxidation via interfacial ⋅ OH radicals, depends on the molecular species adsorbed on the TiO_2_ surface; when the molar ratio between water and methanol adsorbed on the surface of TiO_2_ is above about 300, indirect oxidation by ⋅ OH radicals is the mechanism for photooxidation of methanol by TiO_2_ as suggested by the SFG results.^[92]^

## 7 Aspects that Influence Performance of the Photocatalysts

### 7.1 Properties of the Semiconductors

The intrinsic properties of semiconductors, such as bandgap, nature of the electronic transition (n type or p type) and rate of electron‐hole recombination, have direct influences on the light‐harvesting and efficiency of photocatalytic reactions.[^35^, ^47^, ^97^, ^98^, ^99^, ^100^, ^101^] For example, two well‐known polymorphs of TiO_2_, namely anatase and rutile, have big differences in their intrinsic semiconductor properties even though they have the same chemical composition. It was found that Au particles of similar size on anatase nanoparticles exhibited a rate two orders of magnitude higher than that recorded for Au on rutile nanoparticles in light‐driven ethanol splitting;^[69]^ this can be mainly attributed to that rutile has higher rate of electron–hole recombination than anatase varying between one and over three orders of magnitude depending on the analytical methods.[^98^, ^99^, ^100^, ^101^]

Other properties of semiconductors, such as crystallinity,[^52^, ^102^, ^103^] exposed facets,[^104^, ^105^] and specific surface area^[106]^ also influence the photocatalytic performance. Notably, for an up‐hill reaction, recombination of photogenerated electrons and holes become predominant; in this case, high degree of crystallinity is usually more important than the specific surface area.[^52^, ^103^]

### 7.2 Properties of the Metallic Co‐catalysts

**Figure 8:**
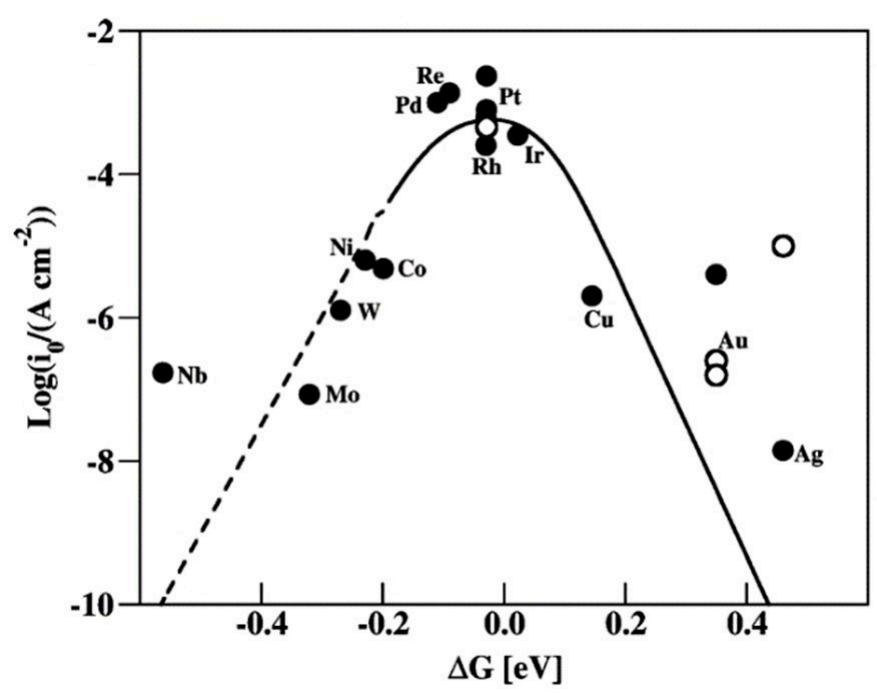
A volcano‐type plot. Measured exchange current density plotted versus the calculated free energy of H adsorption at U=0 V. The open circles are (111) data whereas the filled circles are data from polycrystals. Reproduced with permission from ref. [111]. (Copyright 2010, American Chemical Society).

For LDAS reaction, a key role of metallic co‐catalysts is to collect hydrogen atoms via reducing H^+^ and releasing them as H_2_ molecule, therefore, the electronic properties of the metal are expected to have significant influence on the hydrogen evolution reaction.[^107^, ^108^, ^109^, ^110^, ^111^] Through calculations by a density functional theory, Nørskov and co‐workers find that the most important parameter for describing the hydrogen evolution activity of a metallic electrode is its binding free energy of H. As shown in Figure 8, a volcano‐type curve is obtained when measured activity (exchange current density) is plotted as a function of the calculated free energy of H adsorption.[^110^, ^111^] One can see that too strong or too weak for hydrogen bonding to the metal are unfavourable for the hydrogen evolution reaction; optimum activity is obtained when the interaction is moderate (e. g. Pt).

It has been well‐known that the structural properties of metallic catalysts, for example, size,[^3^, ^112^, ^113^, ^114^] surface structure,[^115^, ^116^, ^117^, ^118^, ^119^, ^120^] play key roles in the catalytic performance. Hydrogenation and hydrogenolysis reactions can be conducted at room temperature owing to their exothermic nature;[^113^, ^121^, ^122^, ^123^] besides, a key intermediate for hydrogenation and hydrogenolysis is M−H species which has also been proposed to exist during LDAS process,[^29^, ^65^, ^74^] therefore, the hydrogenation and hydrogenolysis reactions are expected to occur in LDAS.^[74]^ When these side reactions are taken into account, structural properties of the metallic co‐catalysts could determine the distribution of the final products (i. e. selectivity) in LDAS. Ruberu and co‐workers reported Pt‐modified CdS favors dehydrogenation over hydrogenolysis (benzaldehyde as main product) whereas Pd‐modified CdS favors hydrogenolysis over dehydrogenation (toluene as main product) in light‐driven splitting of benzyl alcohol.^[74]^ Given the similar size of the Pt and Pd NPs, this may be related to the nature of the metals, presumably their electronic properties.

**Figure 9:**
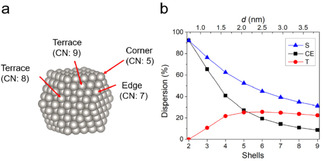
(a) An atomic model of a cuboctahedral cluster (fcc structure) with 5 compete shells. CN: coordination number. (b) Dispersion values as a function of complete shells (bottom axis) and estimated diameters (top axis) of the clusters. S: total surface atoms, CE: corner and edge atoms, T: terrace atoms.

Structure‐performance relationships gained from conventional hydrogenation and hydrogenolysis over supported metal catalysts may also apply in LDAS reaction. Veisz and co‐workers studied the structure sensitivity of the liquid‐phase hydrogenation of styrene to ethylbenzene by Pd‐loaded montmorillonite which was conducted at room temperature (298 K) with a H_2_ pressure of 230 kPa. They prepared Pd nanoparticles with sizes ranging from 1.5 to 6.2 nm by a chemical colloid method and found the shape of the Pd nanoparticles was a cuboctahedron.^[113]^ Figure 9a is an atomic model of a cuboctahedral cluster with 5 compete shells (fcc structure), the surface atoms have different coordination numbers (CN), implying that they may have different catalytic properties. Basing on the cuboctahedral model, number of surface atoms and total number of atoms in the cluster can be calculated according to previously reported formulae;^[124]^ dispersion values for different types of surface sites, calculated via dividing number of the surface sites by total number of atoms in the cluster, were plotted against the number of complete shells and corresponding estimated diameters (*d*) of the clusters (Figure 9b). One can see that the dispersion values for corner and edge atoms (CE), terrace atoms (T) and total surface atoms (S) have different relationships with the estimated diameters of the clusters. TOF will be constant when the correct type of active sites is chosen for the calculations. Veisz and co‐workers reported that low‐coordination surface sites (corner and edge atoms) of Pd nanoparticles are active sites for the hydrogenation of styrene because TOF values are constant only when corner and edge atoms are considered to be the active sites.^[113]^ Notably, the above reaction conditions (298 K, $P_{H_{2}}$ =230 kPa) are similar to those of LDAS reaction albeit that the pressure of H_2_ during LDAS is usually ambient pressure or lower. This implies that the above‐mentioned structure–performance relationship may also apply in LDAS catalyzed by Pd‐modified semiconductors.

**Figure 10:**
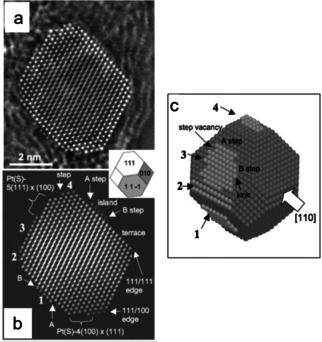
(a) Restored phase of a Pt nanoparticle obtained by employing spherical aberration correction and through‐focus exit wavefunction restoration. (b) Best‐fitting simulated phase. (c) Best‐fitting atomic model. The large white arrow represents the direction of the electron beam. Reproduced with permission from ref. [128] (Copyright 2007, Wiley‐VCH).

In fact, metallic catalysts usually have irregular shapes and their surface structures are far more complicated than those of polyhedral models. Recent developments in spherical‐aberration‐corrected transmission electron microscopy (TEM) and scanning TEM (STEM),[^125^, ^126^] provide opportunities for characterizing materials on the atomic level.[^127^, ^128^, ^129^, ^130^, ^131^, ^132^, ^133^] As shown in Figure 10, imaging surface sites of a Pt nanoparticle supported on carbon black has been realized by applying aberration correction and exit wavefunction restoration.^[128]^ In contrast to the atomic arrangements of the surface of polyhedral models, the outermost atomic layers of the model shown in Figure 10c consist of irregular islands of atoms. These monatomic steps (A type or B type), kinks and vacancies are expected to have different catalytic properties.

Another important aspect is whether there are surfactants or ligands tightly bound on the surfaces of metal NPs. These species are mainly from synthesis of the metal NPs. For example, poly(vinylpyrrolidone) (PVP) is widely used as a capping agent to stabilize small nanoparticles.^[134]^ Previous reports have shown that molecules tightly bound on the metal surfaces are usually unfavorable for the catalytic performance;[^135^, ^136^] although sometimes selectivity of a product is increased, conversion of the reactant is often decreased.[^137^, ^138^, ^139^, ^140^]

### 7.3 Metal/Semiconductor Interfaces

The Schottky barrier formed at the metal/semiconductor interface can suppress the recombination of photogenerated electron‐hole pairs, thus increasing the activity of the photocatalysts.^[46]^ Furthermore, it can prevent the semiconductors (especially for metal chalcogenide‐based semiconductors) from being etched by photocorrosion.[^29^, ^30^, ^74^]

The reverse spillover, that is, hydrogen species migrate from the semiconductor surface to the metal particles where electrons are enriched, and then released as H_2_ molecule by the metal, may become the rate‐limiting step if the density of metal particles on the semiconductor surface is lower than a threshold value.^[75]^ Increasing coverage of metal particles could increase the activity of LDAS as long as the interparticle distance is larger than the Debye length.^[54]^ In addition, strong metal‐support interactions could also be expected,[^141^, ^142^, ^143^, ^144^, ^145^, ^146^, ^147^] especially when the metal particles are loaded on the semiconductors via impregnation and subsequent reduction at elevated temperatures.

Notably, methods utilized for loading co‐catalysts on the semiconductors also have significant influences on the activity of the photocatalysts. Metal‐modified semiconductor photocatalysts prepared by photodeposition methods usually have higher performance than those prepared by impregnation methods.[^31^, ^49^] This can be attributed to the following aspects: the metal particles are loaded on the surface of semiconductors in a random manner for impregnation methods, whereas in the case of photodeposition methods, the metal particles are usually deposited on the surfaces where the electrons are accumulated,[^49^, ^105^, ^148^] benefitting the photocatalytic reactions.

## 8 Conclusion and Outlook

Owing to excellent selectivity for the carbonyl compounds, extremely mild reaction conditions, and easy separation of the photocatalysts from the reaction mixtures, LDAS by heterogeneous photocatalysis has been regarded as a promising route for both selective chemical transformations and sustainable hydrogen production. In this Review, developments in the photocatalysts for LDAS have been discussed. More importantly, two possible mechanisms for LDAS reactions catalyzed by heterogeneous photocatalysts have been proposed on the basis of insights from mechanistic studies.

Although several key advances have been made in this field during the past decade, there are still several issues that need to be addressed.

1. Whether the metallic co‐catalysts are directly involved in the cleavage of the α C−H bond of the alcohol is still unclear. Therefore, more mechanistic studies including in situ tracking the reaction process and DFT calculations are desired.
2. Achieving high chemo‐selectivity in LDAS remains challenge. For instance, converting glycerol, a by‐product in manufacture of bio‐diesel, to value‐added products is of great significance in chemical industry due to the increasing glycerol supply.[^149^, ^150^, ^151^] Light‐driven splitting of glycerol is a promising way for such transformation. Given the two different kinds of OH groups in the glycerol molecule, precisely designing the structures of the photocatalysts and controlling the reaction conditions are required to achieve high chemo‐selectivity. Besides, investigating influence of other functional groups in the alcohol molecules on the LDAS reactions is crucial in order to broadly apply LDAS in organic synthesis.
3. To obtain structure‐performance relationships for the LDAS reactions, deep understanding of the structures of photocatalysts are essential. In particular, knowing surface structures of both the co‐catalysts and the semiconductors on the atomic level are vital for understanding the alcohol adsorption and subsequent transformations.

An emerging research area is to design co‐catalysts for LDAS. For example, structures and properties of the co‐catalysts determine both the hydrogen evolution reaction and the possible side‐reactions. It has been well‐known that different kinds of metals can form solid solutions,[^115^, ^134^, ^152^] intermetallic compounds,[^127^, ^133^, ^134^, ^153^, ^154^] and versatile nanostructures (e. g. core‐shell structures).[^31^, ^134^, ^155^] Investigating structures of the co‐catalysts as well as the metal/semiconductor interfaces on the atomic level utilizing spherical‐aberration‐corrected TEM and scanning TEM (STEM) will certainly create more exciting results.

**Figure 11:**
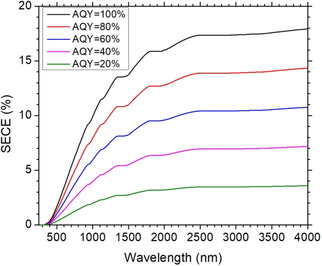
Relationship between calculated SECE and maximum wavelengths of photons which can be utilized by the photocatalysts for splitting of 2‐propanol at different AQYs covering a typical range from 20 to 100%. The calculation was based on standard solar irradiation (ASTM‐G173 AM 1.5 global tilt) and an assumption that one photon can produce one molecular H_2_.

Alcohol splitting driven by sunlight could contribute to a sustainable society. High SECE is extremely desired in this regard. For a specific alcohol splitting reaction, the maximum SECE can be calculated based on standard solar irradiation (ASTM‐G173 AM 1.5 global tilt) and Gibbs free energy change of the reaction. Figure 11 demonstrates relationship between SECE and maximum wavelengths of photons which can be utilized by the photocatalysts for splitting of 2‐propanol at different AQYs. A maximum value of SECE, about 18%, can be achieved by a photocatalyst with an absorption edge around 4000 nm (corresponding to a bandgap of 0.31 eV) with an AQY of 100%. This is an exciting result albeit that a photocatalyst with such a small bandgap has not been reported. To achieve a SECE of 10%, the absorption edges of the photocatalysts are requried to be 1007, 1237, 2200 nm when AQYs are 100%, 80%, 60%, respectively; the corresponding bandgaps of the photocatalysts were calculated to be 1.2, 1.0, 0.56 eV, respectively. Therefore, developing narrow‐bandgap photocatalysts with high AQYs for LDAS reactions is the key aspect to achieve high SECE.

LDAS by heterogeneous photocatalysis has shown great potential in sustainable chemical industry and hydrogen production. To satisfy the requirements of large‐scale applications of LDAS catalyzed by heterogeneous photocatalysts, it is neccessary to design environmentally friendly, efficient, durable and low‐cost photocatalysts. In addition, applying other advanced technologies (such as photovoltaic‐assisted electrolysis, electrocatalytic conversions and photothermal catalysis) in this research field will stimulate new discoveries, providing more opportunities for realizing industrial applications of LDAS.

## Conflict of interest

The author declares no conflict of interest.

## Biographical Information

*Zhigang Chai studied physical chemistry at the Peking University and received his Ph.D. in 2016. He then continued his scientific education as a post‐doctoral fellow at Uppsala University, Sweden. He is now a researcher at Uppsala University. His current research interest is developing heterogeneous photocatalysts for solar‐fuels and selective chemical transformations*.